# 2-[(4-Chloro­benzo­yl)­hydrazono]­propionic acid monohydrate

**DOI:** 10.1107/S1600536809009544

**Published:** 2009-03-25

**Authors:** Hon Wee Wong, Kong Mun Lo, Seik Weng Ng

**Affiliations:** a Department of Chemistry, University of Malaya, 50603 Kuala Lumpur, Malaysia

## Abstract

In the title compound, C_10_H_9_ClN_2_O_3_·H_2_O, the water mol­ecule is a hydrogen-bond donor to the amide and carbonyl O atoms of two acid mol­ecules; it is also a hydrogen-bond acceptor to the acid OH group and the amide H atom. The hydrogen-bonding inter­actions give rise to a two-dimensional array.

## Related literature

For the structure of 2-[(4-methyl­benzo­yl)hydrazono]propionic acid monohydrate, see: Wong *et al.* (2009 ▶).
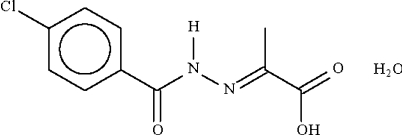


## Experimental

### 

#### Crystal data

C_10_H_9_ClN_2_O_3_·H_2_O
*M* *_r_* = 258.66
Triclinic, 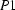

*a* = 6.6516 (1) Å
*b* = 6.9345 (1) Å
*c* = 7.0988 (1) Å
α = 73.833 (1)°
β = 80.182 (1)°
γ = 61.613 (1)°
*V* = 276.39 (1) Å^3^
*Z* = 1
Mo *K*α radiation
μ = 0.35 mm^−1^
*T* = 118 K
0.45 × 0.35 × 0.15 mm

#### Data collection

Bruker SMART APEX diffractometer
Absorption correction: multi-scan (*SADABS*; Sheldrick, 1996 ▶) *T*_min_ = 0.858, *T*_max_ = 0.949
2247 measured reflections
1965 independent reflections
1952 reflections with *I* > 2σ(*I*)
*R*_int_ = 0.011

#### Refinement

*R*[*F* ^2^ > 2σ(*F* ^2^)] = 0.026
*wR*(*F* ^2^) = 0.070
*S* = 1.00
1965 reflections
171 parameters
7 restraints
H atoms treated by a mixture of independent and constrained refinement
Δρ_max_ = 0.20 e Å^−3^
Δρ_min_ = −0.33 e Å^−3^
Absolute structure: Flack (1983 ▶), 733 Friedel pairs
Flack parameter: 0.02 (3)

### 

Data collection: *APEX2* (Bruker, 2008 ▶); cell refinement: *SAINT* (Bruker, 2008 ▶); data reduction: *SAINT*; program(s) used to solve structure: *SHELXS97* (Sheldrick, 2008 ▶); program(s) used to refine structure: *SHELXL97* (Sheldrick, 2008 ▶); molecular graphics: *X-SEED* (Barbour, 2001 ▶); software used to prepare material for publication: *pubCIF* (Westrip, 2009 ▶).

## Supplementary Material

Crystal structure: contains datablocks global, I. DOI: 10.1107/S1600536809009544/tk2398sup1.cif

Structure factors: contains datablocks I. DOI: 10.1107/S1600536809009544/tk2398Isup2.hkl

Additional supplementary materials:  crystallographic information; 3D view; checkCIF report

## Figures and Tables

**Table 1:** Hydrogen-bond geometry (Å, °)

*D*—H⋯*A*	*D*—H	H⋯*A*	*D*⋯*A*	*D*—H⋯*A*
O1—H1O⋯O1*W*	0.83 (1)	1.92 (3)	2.659 (2)	147 (4)
O1*W*—H11⋯O2^i^	0.84 (1)	1.96 (1)	2.784 (2)	165 (2)
O1*W*—H12⋯O3	0.84 (1)	1.98 (1)	2.809 (2)	172 (2)
N1—H1N⋯O1*W*^ii^	0.88 (1)	2.48 (2)	3.3596 (18)	177 (2)

Symmetry codes: (i) 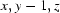; (ii) 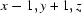.

**Fig. 1.**
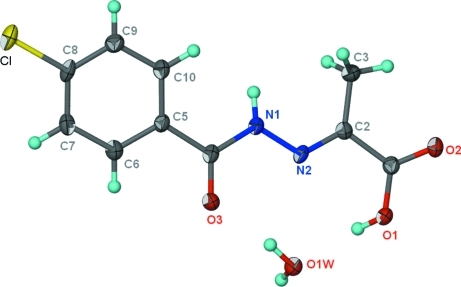
Thermal ellipsoid plot (Barbour, 2001) of C10H9N2O3.H2O at the 70% probability level. Hydrogen atoms are drawn as spheres of arbitrary radius.

## supplementary crystallographic information

### Experimental

4-Chlorobenzoylhydrazide (0.85 g, 0.005 mol) and pyruvic acid (0.43 g, 0.005 mol) were dissolved in methanol (30 ml). The solution was heated for 3 h; slow evaporation of the solvent gave colorless crystals.

### Refinement

Carbon-bound H-atoms were placed in calculated positions (C–H 0.95-0.98 Å) and were included in the refinement in the riding model approximation, with *U*(H) set to 1.2-1.5*U*(C). The methyl H-atoms were rotated to fit the electron density.

The oxygen- and nitrogen-bound H-atoms were located in a difference Fourier map, and were refined with distance restraints [N–H 0.88±0.01 and O–H 0.84±0.01 Å]; their U_iso_ values were freely refined.

### Crystal data

**Table:**

C_10_H_9_ClN_2_O_3_·H_2_O	*Z* = 1
*M**_r_* = 258.66	*F*(000) = 134
Triclinic, *P*1	*D*_x_ = 1.554 Mg m^−^^3^
Hall symbol: P 1	Mo *K*α radiation, λ = 0.71073 Å
*a* = 6.6516 (1) Å	Cell parameters from 2199 reflections
*b* = 6.9345 (1) Å	θ = 3.0–28.2°
*c* = 7.0988 (1) Å	µ = 0.35 mm^−^^1^
α = 73.833 (1)°	*T* = 118 K
β = 80.182 (1)°	Irregular block, colorless
γ = 61.613 (1)°	0.45 × 0.35 × 0.15 mm
*V* = 276.39 (1) Å^3^	

### Data collection

**Table:**

Bruker SMART APEX diffractometer	1965 independent reflections
Radiation source: fine-focus sealed tube	1952 reflections with *I* > 2σ(*I*)
graphite	*R*_int_ = 0.011
ω scans	θ_max_ = 27.5°, θ_min_ = 3.0°
Absorption correction: multi-scan (*SADABS*; Sheldrick, 1996)	*h* = −8→8
*T*_min_ = 0.858, *T*_max_ = 0.949	*k* = −8→8
2247 measured reflections	*l* = −9→9

### Refinement

**Table:**

Refinement on *F*^2^	Secondary atom site location: difference Fourier map
Least-squares matrix: full	Hydrogen site location: inferred from neighbouring sites
*R*[*F*^2^ > 2σ(*F*^2^)] = 0.026	H atoms treated by a mixture of independent and constrained refinement
*wR*(*F*^2^) = 0.070	*w* = 1/[σ^2^(*F*_o_^2^) + (0.0628*P*)^2^ + 0.0013*P*] where *P* = (*F*_o_^2^ + 2*F*_c_^2^)/3
*S* = 1.00	(Δ/σ)_max_ < 0.001
1965 reflections	Δρ_max_ = 0.20 e Å^−^^3^
171 parameters	Δρ_min_ = −0.33 e Å^−^^3^
7 restraints	Absolute structure: Flack (1983), 733 Friedel pairs
Primary atom site location: structure-invariant direct methods	Flack parameter: 0.02 (3)

### Fractional atomic coordinates and isotropic or equivalent isotropic displacement parameters (Å^2^)

**Table:**

	*x*	*y*	*z*	*U*_iso_*/*U*_eq_	
Cl1	0.50015 (5)	0.49992 (5)	0.49993 (4)	0.02415 (12)	
O1	2.0776 (2)	0.41172 (18)	−0.23906 (16)	0.0184 (2)	
O2	1.9975 (2)	0.76916 (19)	−0.31287 (18)	0.0221 (3)	
O3	1.5700 (2)	0.18182 (19)	0.11932 (19)	0.0228 (3)	
O1W	1.9830 (2)	0.06405 (18)	−0.10712 (18)	0.0216 (2)	
N1	1.4403 (2)	0.5584 (2)	−0.0003 (2)	0.0168 (3)	
N2	1.6543 (2)	0.5201 (2)	−0.07703 (19)	0.0154 (3)	
C1	1.9322 (3)	0.6250 (3)	−0.2491 (2)	0.0161 (3)	
C2	1.6887 (3)	0.6861 (2)	−0.1806 (2)	0.0165 (3)	
C3	1.5134 (3)	0.9273 (3)	−0.2314 (3)	0.0279 (4)	
H3A	1.3862	0.9390	−0.2937	0.042*	
H3B	1.5830	1.0164	−0.3222	0.042*	
H3C	1.4562	0.9845	−0.1115	0.042*	
C4	1.4131 (3)	0.3718 (3)	0.1048 (2)	0.0169 (3)	
C5	1.1830 (3)	0.4130 (3)	0.2013 (2)	0.0158 (3)	
C6	1.1356 (3)	0.2284 (3)	0.2676 (2)	0.0197 (3)	
H6	1.2483	0.0845	0.2492	0.024*	
C7	0.9270 (3)	0.2526 (3)	0.3596 (2)	0.0200 (3)	
H7	0.8955	0.1268	0.4045	0.024*	
C8	0.7637 (3)	0.4649 (3)	0.3852 (2)	0.0187 (3)	
C9	0.8072 (3)	0.6496 (3)	0.3209 (2)	0.0194 (3)	
H9	0.6934	0.7934	0.3385	0.023*	
C10	1.0168 (3)	0.6238 (2)	0.2310 (2)	0.0184 (3)	
H10	1.0483	0.7497	0.1891	0.022*	
H1O	2.032 (8)	0.321 (6)	−0.244 (7)	0.115 (18)*	
H11	1.970 (4)	−0.004 (3)	−0.182 (3)	0.029 (5)*	
H12	1.855 (2)	0.111 (4)	−0.048 (3)	0.028 (6)*	
H1N	1.318 (3)	0.689 (2)	−0.024 (3)	0.013 (4)*	

### Atomic displacement parameters (Å^2^)

**Table:**

	*U*^11^	*U*^22^	*U*^33^	*U*^12^	*U*^13^	*U*^23^
Cl1	0.0181 (2)	0.0375 (2)	0.02137 (18)	−0.01711 (17)	0.00484 (13)	−0.00835 (14)
O1	0.0136 (6)	0.0172 (5)	0.0225 (6)	−0.0068 (4)	0.0026 (4)	−0.0041 (4)
O2	0.0191 (6)	0.0202 (5)	0.0283 (6)	−0.0120 (5)	0.0068 (5)	−0.0066 (4)
O3	0.0183 (6)	0.0153 (5)	0.0309 (6)	−0.0077 (4)	0.0049 (5)	−0.0028 (4)
O1W	0.0183 (6)	0.0182 (5)	0.0274 (6)	−0.0087 (5)	0.0051 (5)	−0.0067 (4)
N1	0.0140 (7)	0.0163 (6)	0.0188 (6)	−0.0074 (5)	0.0021 (5)	−0.0027 (5)
N2	0.0120 (7)	0.0196 (6)	0.0160 (6)	−0.0082 (5)	0.0032 (5)	−0.0064 (5)
C1	0.0167 (8)	0.0171 (6)	0.0156 (6)	−0.0087 (6)	0.0011 (6)	−0.0044 (5)
C2	0.0165 (8)	0.0158 (7)	0.0176 (7)	−0.0080 (6)	0.0008 (6)	−0.0038 (5)
C3	0.0177 (8)	0.0165 (7)	0.0415 (10)	−0.0060 (6)	0.0068 (7)	−0.0028 (6)
C4	0.0154 (8)	0.0191 (7)	0.0173 (7)	−0.0093 (6)	0.0014 (6)	−0.0044 (5)
C5	0.0137 (8)	0.0178 (7)	0.0153 (7)	−0.0081 (6)	0.0014 (6)	−0.0026 (5)
C6	0.0197 (8)	0.0195 (7)	0.0206 (7)	−0.0105 (6)	0.0010 (6)	−0.0036 (5)
C7	0.0213 (9)	0.0222 (7)	0.0212 (7)	−0.0147 (7)	0.0018 (6)	−0.0044 (6)
C8	0.0143 (8)	0.0279 (8)	0.0151 (7)	−0.0123 (7)	0.0019 (6)	−0.0030 (6)
C9	0.0168 (8)	0.0191 (7)	0.0182 (7)	−0.0064 (6)	−0.0003 (6)	−0.0016 (5)
C10	0.0176 (8)	0.0187 (7)	0.0185 (7)	−0.0098 (6)	0.0018 (6)	−0.0023 (5)

### Geometric parameters (Å, °)

**Table:**

Cl1—C8	1.7363 (18)	C3—H3B	0.9800
O1—C1	1.3161 (19)	C3—H3C	0.9800
O1—H1O	0.829 (10)	C4—C5	1.493 (2)
O2—C1	1.219 (2)	C5—C6	1.399 (2)
O3—C4	1.220 (2)	C5—C10	1.400 (2)
O1W—H11	0.842 (9)	C6—C7	1.382 (2)
O1W—H12	0.836 (10)	C6—H6	0.9500
N1—N2	1.360 (2)	C7—C8	1.393 (2)
N1—C4	1.379 (2)	C7—H7	0.9500
N1—H1N	0.878 (9)	C8—C9	1.383 (2)
N2—C2	1.281 (2)	C9—C10	1.380 (3)
C1—C2	1.495 (2)	C9—H9	0.9500
C2—C3	1.497 (2)	C10—H10	0.9500
C3—H3A	0.9800		
			
C1—O1—H1O	120 (3)	N1—C4—C5	116.93 (13)
H11—O1W—H12	103 (2)	C6—C5—C10	119.14 (15)
N2—N1—C4	116.41 (12)	C6—C5—C4	117.42 (14)
N2—N1—H1N	124.6 (14)	C10—C5—C4	123.44 (14)
C4—N1—H1N	118.6 (14)	C7—C6—C5	120.85 (14)
C2—N2—N1	119.24 (13)	C7—C6—H6	119.6
O2—C1—O1	119.90 (16)	C5—C6—H6	119.6
O2—C1—C2	121.04 (14)	C6—C7—C8	118.77 (14)
O1—C1—C2	119.06 (13)	C6—C7—H7	120.6
N2—C2—C1	114.38 (13)	C8—C7—H7	120.6
N2—C2—C3	126.56 (16)	C9—C8—C7	121.30 (17)
C1—C2—C3	119.03 (14)	C9—C8—Cl1	119.04 (13)
C2—C3—H3A	109.5	C7—C8—Cl1	119.67 (13)
C2—C3—H3B	109.5	C10—C9—C8	119.64 (15)
H3A—C3—H3B	109.5	C10—C9—H9	120.2
C2—C3—H3C	109.5	C8—C9—H9	120.2
H3A—C3—H3C	109.5	C9—C10—C5	120.28 (14)
H3B—C3—H3C	109.5	C9—C10—H10	119.9
O3—C4—N1	121.63 (16)	C5—C10—H10	119.9
O3—C4—C5	121.44 (15)		
			
C4—N1—N2—C2	178.11 (13)	N1—C4—C5—C10	−16.0 (2)
N1—N2—C2—C1	177.31 (11)	C10—C5—C6—C7	0.7 (2)
N1—N2—C2—C3	−0.7 (2)	C4—C5—C6—C7	179.58 (13)
O2—C1—C2—N2	−164.68 (14)	C5—C6—C7—C8	0.0 (2)
O1—C1—C2—N2	15.00 (19)	C6—C7—C8—C9	−0.1 (2)
O2—C1—C2—C3	13.5 (2)	C6—C7—C8—Cl1	179.90 (11)
O1—C1—C2—C3	−166.78 (14)	C7—C8—C9—C10	−0.6 (2)
N2—N1—C4—O3	−3.7 (2)	Cl1—C8—C9—C10	179.43 (11)
N2—N1—C4—C5	175.94 (11)	C8—C9—C10—C5	1.3 (2)
O3—C4—C5—C6	−15.2 (2)	C6—C5—C10—C9	−1.3 (2)
N1—C4—C5—C6	165.12 (13)	C4—C5—C10—C9	179.82 (13)
O3—C4—C5—C10	163.61 (15)		

### Hydrogen-bond geometry (Å, °)

**Table:**

*D*—H···*A*	*D*—H	H···*A*	*D*···*A*	*D*—H···*A*
O1—H1O···O1W	0.83 (1)	1.92 (3)	2.659 (2)	147 (4)
O1W—H11···O2^i^	0.84 (1)	1.96 (1)	2.784 (2)	165 (2)
O1W—H12···O3	0.84 (1)	1.98 (1)	2.809 (2)	172 (2)
N1—H1N···O1W^ii^	0.88 (1)	2.48 (2)	3.3596 (18)	177 (2)

Symmetry codes: (i) *x*, *y*−1, *z*; (ii) *x*−1, *y*+1, *z*.
